# Diversity of MSDIN family members in amanitin-producing mushrooms and the phylogeny of the MSDIN and prolyl oligopeptidase genes

**DOI:** 10.1186/s12864-020-06857-8

**Published:** 2020-06-26

**Authors:** Zhengmi He, Pan Long, Fang Fang, Sainan Li, Ping Zhang, Zuohong Chen

**Affiliations:** College of Life Science, Hunan Normal University, Lushan Road, Changsha, 410081 China

**Keywords:** *Amanita*, *Galerina*, *Lepiota*, Cyclopeptide toxin, Prolyl oligopeptidase, Horizontal gene transfer

## Abstract

**Background:**

Amanitin-producing mushrooms, mainly distributed in the genera *Amanita*, *Galerina* and *Lepiota*, possess MSDIN gene family for the biosynthesis of many cyclopeptides catalysed by prolyl oligopeptidase (POP). Recently, transcriptome sequencing has proven to be an efficient way to mine MSDIN and *POP* genes in these lethal mushrooms. Thus far, only *A*. *palloides* and *A. bisporigera* from North America and *A*. *exitialis* and *A. rimosa* from Asia have been studied based on transcriptome analysis. However, the MSDIN and *POP* genes of many amanitin-producing mushrooms in China remain unstudied; hence, the transcriptomes of these speices deserve to be analysed.

**Results:**

In this study, the MSDIN and *POP* genes from ten *Amanita* species, two *Galerina* species and *Lepiota venenata* were studied and the phylogenetic relationships of their MSDIN and *POP* genes were analysed. Through transcriptome sequencing and PCR cloning, 19 *POP* genes and 151 MSDIN genes predicted to encode 98 non-duplicated cyclopeptides, including α-amanitin, β-amanitin, phallacidin, phalloidin and 94 unknown peptides, were found in these species. Phylogenetic analysis showed that (1) MSDIN genes generally clustered depending on the taxonomy of the genus, while *Amanita* MSDIN genes clustered depending on the chemical substance; and (2) the *POPA* genes of *Amanita*, *Galerina* and *Lepiota* clustered and were separated into three different groups, but the *POPB* genes of the three distinct genera were clustered in a highly supported monophyletic group.

**Conclusions:**

These results indicate that lethal *Amanita* species have the genetic capacity to produce numerous cyclopeptides, most of which are unknown, while lethal *Galerina* and *Lepiota* species seem to only have the genetic capacity to produce α-amanitin. Additionally, the *POPB* phylogeny of *Amanita*, *Galerina* and *Lepiota* conflicts with the taxonomic status of the three genera, suggesting that underlying horizontal gene transfer has occurred among these three genera.

## Background

Amatoxins, which are lethal substances found in mushrooms, have mainly been reported to be present in species from three distinct genera classified into three different families: *Amanita* (Amanitaceae), *Galerina* (Hymenogastraceae) and *Lepiota* (Agaricaceae) [1–4]. Among these amanitin-producing mushrooms, lethal *Amanita* species are the best-known and most typical mushrooms that produce three primary groups of cyclopeptide toxins: amatoxins, phallotoxins and virotoxins, which are bicyclic octapeptides, bicyclic heptapeptides and monocyclic heptapeptides, respectively [1–4].

The precursor peptide genes of α-amanitin (α-AMA) and phallacidin (PHD) along with multiple related sequences encoding unknown cyclic peptides were first identified and predicted in *Amanita bisporigera* by genome shotgun sequencing, indicating that amatoxins and phallotoxins are encoded by the same gene family and are biosynthesized on ribosomes [5]. This gene family is referred to as MSDIN in reference to the first five conserved encoded amino acids, and the precursor peptides of its members contain 33–37 amino acids, consisting of two conserved regions, 10 upstream amino acids and 17 downstream amino acids, a highly variable core region, and a 6–10 amino acid sequence that ultimately forms the corresponding cyclopeptide [6]. *GmAMA*, which is responsible for producing α-AMA, is also found in the genome of *Galerina marginata* [7]. *Galerina marginata* is a specific amanitin-containing species in the genus *Galerina*. Unlike lethal amanitas, *G. marginata* does not harbour MSDIN-like family genes other than two copies of *α-AMA* genes. Additionally, *α-AMA* of *Lepiota brunneoincarnata*, which is an amanitin-containing mushroom of the genus *Lepiota*, has been successfully cloned [8]. The genome sequencing of *L*. *venenata*, another newly reported amanitin-containing species, has been completed, and it has been shown to harbour *α-AMA* genes [9]. Precursor peptide sequence alignment of *α-AMA* sequences from *Amanita*, *Galerina* and *Lepiota* shows high divergence except in the toxin region.

It has been strongly indicated that a prolyl oligopeptidase (POP) plays an important role in the initial processing of MSDIN precursor peptides. Since the core toxin regions are flanked by two highly conserved proline (Pro) residues, this enzyme can cleave the C-terminus of Pro residues and release the peptide chain of the toxin to form a cyclopeptide [10]. It has been reported that there are two types of POP in amatoxin-producing mushrooms: POPA, which behaves like a conventional housekeeping protein that is present in all species, and POPB, which is the enzyme that actually catalyses the cutting and cyclization of precursor peptides [7, 11, 12].

Increasing numbers of MSDIN family members have been published since the first 15 MSDIN genes were found in the *A. bisporigera* genome, and four were amplified by using degenerate primers in *A. phalloides* and *A. ocreata* [5]. Twenty-four MSDIN members were obtained from 6 *Amanita* species using degenerate primers [13]. Recently, the draft genome sequences of *A*. *palloides* and *A. bisporigera* showed that each species possessed approximately 30 MSDIN members, but only three of these genes were common to the two fungi [6]. Eighteen and twenty-two MSDIN genes were mined from the *A*. *subjunquillea* and *A*. *pallidorosea* genomes through PacBio and Illumina sequencing, respectively [8]. However, the MSDIN genes of many amanitin-containing *Amanita*, *Galerina* and *Lepiota* mushrooms have not been investigated in depth to date. Lethal *Amanita* species are classified in section *Phalloideae* of the genus *Amanita* [14, 15]. Approximately 50 lethal *Amanita* species have been reported worldwide, and the species diversity of lethal amanitas is strongly underestimated under the current taxonomy [15, 16]. Many new lethal *Amanita* and *Lepiota* species, including *A. rimosa*, *A*. *subfuliginea*, *A*. *subpallidorosea,* and *L. venenata*, have been discovered over the past decade [17–20]. In addition to the 22 known cyclopeptide toxins, some new cyclopeptide substances, such as cycloamanide E and cycloamanide F in *A. phalloides* and amanexitide in *A. exitialis*, have been extracted and identified [6, 21, 22]. It has been reported that *A. bisporigera* and *A. phalloides* present high potential for the biosynthesis of a variety of cyclopeptides, most of which are unknown according to predictions. Hence, considering the species diversity of amanitin-containing mushrooms and the broad genetic capacity of lethal amanitas to produce unknown cyclopeptides, there are still many new cyclopeptide genes and corresponding cyclopeptides to be discovered.

Alpha-amanitin and toxin-biosyntheic prolyl oligopeptidase B (POPB) genes have been proven to exist in some lethal *Amanita* [6, 23, 24], *Galerina* [7] and *Lepiota* [9] species. The reason that the biosynthetic pathway for α-amanitin is present in these three phylogenetically disjunct genera classified in different families has been studied in recent years. Recent studies reported that horizontal gene transfer (HGT) is the underlying cause of the distribution of MSDIN and *POPB* genes in *Amanita*, *Galerina* and *Lepiota* on the basis of phylogenetic analysis [8, 9]. The possibility of convergent evolution was negated because the MSDIN and *POPB* genes in these three genera show similarity and associations, such as a shared conserved gene structure and the encoding of precursor peptides by MSDIN genes [8].

According to previous research, whole-genome sequencing has proven to be the most comprehensive, in-depth method for identifying MSDIN genes or genes related to the cyclopeptide biosynthetic pathway in amanitin-producing mushrooms [6, 8]. Nevertheless, compared with genome sequencing, transcriptome sequencing provides an alternative efficient and low-cost method to obtain functional gene data. To the best of our knowledge, only *A*. *palloides* and *A. bisporigera* from North America and *A*. *exitialis* and *A. rimosa* from Asia have been studied using transcriptome sequencing [6, 25, 26].

In this study, the transcriptomes of seven amanitin-producing mushrooms (*A. exitialis*, *A. fuliginea*, *A. molliuscula*, *A. pallidorosea*, *A. rimosa*, *A. subpallidorosea* and *L. venenata*) and an *Amanita* species producing no amanitin (*A. oberwinklerana*) were sequenced. MSDIN and *POP* genes were searched and predicted from the transcriptome data. The genomic and coding sequences of the MSDIN and *POP* genes were cloned and verified. Similarly, MSDIN and *POP* sequences were cloned from two *Galerina* strains (*G. marginata* and *G*. *sulciceps*). In addition to the *Amanita* species mentioned above, MSDIN genes from *A. subfuliginea*, *A. subjunquillea* and *A. virosa* were cloned using specific and degenerate primers. Furthermore, phylogenetic analysis was performed on the obtained toxin and *POP* genes. Our study was aimed at (a) identifying MSDIN genes from amanitin-producing mushrooms to guide the isolation and identification of new unknown related cyclopeptides and (b) determining the evolutionary relationships of toxin MSDIN and *POP* genes in amanitin-producing mushrooms.

## Results

### Data filtering and assembly of transcriptomes

Transcriptome sequencing of seven amanitin-producing mushrooms was performed on the BGISEQ-500 platform using the combinational probe-anchor synthesis sequencing method. After the removal of ambiguous, adaptor-containing and low-quality sequences, clean data were obtained and de novo assembled using Trinity software. The main transcriptomic features and NCBI accession numbers of the transcriptome data obtained in our study are presented in Table 1.

**Table 1:** Features and accession numbers of transcriptomes

Species	Total clean bases (Gb)	Q30 (%)	Total number of unigene	Total length of unigene (nt)	Mean length of unigene (nt)	N50	GC (%)	accession number
*A. exitialis*	6.67	86.25	24,578	48,383,999	1968	2891	49.75	SRR9929233
*A. fuliginea*	6.55	88.20	21,624	36,817,429	1702	2599	49.54	SRR9937194
*A. molliuscula*	6.32	90.65	46,471	79,566,952	1712	3007	49.71	SRR9937646
*A. oberwinklerana*	6.59	86.87	24,326	61,864,918	2543	3993	48.83	SRR9937816
*A. pallidorosea*	6.25	89.78	36,846	79,216,743	2149	3375	49.52	SRR9937866
*A. rimosa*	6.57	87.93	22,532	36,712,344	1629	2648	49.05	SRR9943992
*A. subpallidorosea*	10.24	91.21	42,803	110,323,057	2577	3630	49.00	SRR9943549
*L. venenata*	8.47	93.83	13,859	21,738,818	1569	2994	48.88	SRR9943552

### MSDIN and *POP* genes

Through transcriptome sequencing, 110 MSDIN genes (Table 2) were manually identified in 7 lethal *Amanita* and *Lepiota* species using known MSDIN members from *A. bisporigera* as TBLASTN queries. Additionally, 70 MSDIN genes (Table 3) were obtained from 12 lethal *Amanita*, *Galerina* and *Lepiota* species by PCR cloning using degenerate and specific primers. In general, a total of 151 nonrepetitive MSDIN genes were identified at the genomic and transcriptomic levels by using these methods. All the obtained MSDIN genes were predicted to encode 98 cyclopeptides, including α-amanitin (IWGIGCNP), β-amanitin (IWGIGCDP), phallacidin (AWLVDCP), phalloidin (AWLATCP) and 94 unknown peptides. These predicted cyclopeptides were composed of 6–11 amino acids and included 5 hexapeptides, 30 heptapeptides, 73 octapeptides, 22 nanopeptides, 19 decapeptides, and 2 undecapeptides.

**Table 2:** MSDIN family members searched from the transcriptomes of seven amanitin-producing mushrooms

Name	No.	Leader peptide	Core peptide	Recognition sequence	Product
*A. exitialis*	Ae1	MTDINDTRLP	**FIWLLWIWLP**	SVGDDNNILNRGEDLC*	
	Ae2	MSDINATRLP	**LFFPPDFRPP**	CVGDADNFTLTRGENLC*	
	Ae3	MSDVNATRLP	**FNFFRFPYP**	CIGDDSGSALRLGESLC*	
	Ae4	MSDINTARLP	**IPVPPFFIP**	FVGDDIDVVLRRGENLC*	
	Ae5	MSDINVTRLP	**VFIFFFIPP**	CVGDGTADIVRKGENLC*	
	Ae6	MSDINTARLP	**VFSLPVFFP**	FVSDDIQAVLTRGESLC*	
	Ae7	MSDINTTRLP	**FVFVASPP**	CVGDDIAMVLTRGENLC*	
	Ae8	MSDINPTRLP	**IFWFIYFP**	CVSDVDSTLTLCISLS*	
	Ae9	MSDINTARLP	**IIWIIGNP**	CVSDDVERILTRGESLC*	
	Ae10	MSDINATRLP	**IIWAPVVP**	CISDDNDSTLTRGQSLC*	
	Ae11	MSDINATRLP	**IGRPQLLP**	CVGGDVNYILISGENLC*	
	Ae12	MSDINATRLP	**IWGIGCDP**	CVGDDVTALLTRGEALC*	β-amanitin
	Ae13	MSDINATRLP	**IWGIGCNP**	CVGDDVTSVLTRGEALC*	α-amanitin
	Ae14	MSDINVIRLP	**SMLTILPP**	CVSDDASNTLTRGENLC*	
	Ae15	MSDINATRLP	**AWLTDCP**	CVGDDVNRLLTRGESLC*	“phallotoxin”
	Ae16	MSDINATRLP	**AWLVDCP**	CVGDDVNRLLTRGESLC*	phallacidin
	Ae17	MSDINLTRLP	**GIIAIIP**	CVGDDVNSTLTRGQSLC*	
	Ae18	MSDINATRLP	**VWIGYSP**	CVGDDCIALLTRGEGLC*	
	Ae19	MSDINATRLP	**GFLFWA**	YVGDDVDYILTRGESLA*	
*A. fuliginea*	Af1	MSDINATRLP	**IIIVLGLIIP**	LCVSDIEMILTRGESLC*	
	Af2	MSDLNASRLP	**ILSVLGLPVP**	HVGEETNSTLARGESLC*	
	Af3	MSDINSARLP	**LFFPPIFIPP**	CVSDDVQVVLTRGENLC*	
	Af4	MSDINAARLP	**FFPFVFIPP**	CIGDDATSIVRQAENLC*	
	Af5	MSDINTIRIP	**FPWTGPFVP**	CVSDDVGSVLMRGESLS*	
	Af6	MSDTNATRLP	**IWFIQLQIP**	CAGDDVNSSLTRGESLC*	
	Af7	MSDINVTRLP	**VLVFIFFPP**	YISDDAVNILKQGENLC*	
	Af8	MFDINGSRLP	**AFRLIPPP**	CVGDDVDSTLTSGESLC*	
	Af9	MSDINATRLP	**GILIVFPP**	CVGDDVNSTLTRGESLC*	
	Af10	MSDINATRLP	**HLFTWIPP**	CISDDSTLTRGESFC*	
	Af11	MFDINSSRLP	**HLYPNSRP**	CVCDDACSTLTSAESLC*	
	Af12	MSDINATRLP	**IFWFIYFP**	CVGDDVDNTLTRGESLS*	
	Af13	MSDINATRLP	**IWGIGCDP**	CVGDDVAALITRGEALC*	β-amanitin
	Af14	MSCINATRLP	**LPSRPVFP**	FVSDAIEVVLGRGEDLC*	
	Af15	MSDINSLRLP	**VVNSRFNP**	CVGDDVSPTLTRGEGLC*	
	Af16	MSDINASRLP	**AWLATCP**	CIGDDVNPTITRGESLC*	phalloidin
	Af17	MSDINATRLP	**AWLVDCP**	CVGDDVNRLLARGENLC*	phallacidin
	Af18	MSDINATRLP	**AWLVTCP**	CVGDDINRLLTRGENLC*	“phallotoxin”
*A. molliuscula*	Am1	MSDINTARLP	**YFLPPIFSPP**	CVSDDIEMVLTRGENLC*	
	Am2	MTDINATRLP	**ILFGFFLLP**	CVDGVDNTLHSGENLC*	
	Am3	MSNINASRLP	**IWAAFFRFP**	CVGDEVDGILRSGESLC*	
	Am4	MSDINATRLA	**IWGIGCDP**	CVGDDVTALLTRGEALC*	β-amanitin
	Am5	MSDINASRLP	**RLLVPRYP**	CIDEDAEGATYLC*	
	Am6	MSNINAIRLP	**GFFAVVP**	YLATSITFSLLGRGESLC*	
	Am7	MTDINATRLP	**WIFFFPP**	CVDDVDNTLHSGENLC*	
	Am8	MSNINALRLP	**GFGFIP**	YASGDVDYTLTRGESLS*	
	Am9	MSDINATRFP	**GKVNPP**	YVGDDVDDIIIRGEKLC*	
*A. pallidorosea*	Ap1	MADINAARLP	**FHGLFPFLPPP**	FVDDDATSTLTRGESLC*	
	Ap2	MADINASRLP	**LNILPFHLPP**	CVSDDATSTLTRGESLC*	
	Ap3	MSDINATRLP	**NWHAGPTRPP**	CVADDVSLTLTRGESLC*	
	Ap4	MSDINTARLP	**VFFMPPFIPP**	CVSDDIQMVLTRGENLC*	
	Ap5	MSDINTARLP	**EFIVFGIFP**	CVGDDIQTVLTRGEDLC*	
	Ap6	MSDINASRLP	**FFPEVGFFP**	CVGDDTNPILTRGGSLS*	
	Ap7	MSDLNATRLP	**FNLFRFPYP**	CIGDDSGSVLTLGEGLC*	
	Ap8	MSDINTIRVP	**FPWTGPFVP**	CVGDDVGSVLTHGESLS*	
	Ap9	MSDINATRLP	**HPFPLGLQP**	CAGDVDNLTLFRGEGLC*	
	Ap10	MSDINATRLP	**DPRRLLIP**	GSSDDVDSALTRGESLC*	
	Ap11	MSDINTTRLP	**HFFNLMPP**	CVGDDIETVLTRGESLC*	
	Ap12	MSDINATRLP	**HQHHPFVP**	GGSDDVGSTLTRGESLC*	
	Ap13	MSDMNVVRLP	**ISDPTAYP**	CVGDDIQAVLGRGESLC*	
	Ap14	MSDINATRLP	**IWGIGCDP**	CVGDDVTAVLTRGEALC*	β-amanitin
	Ap15	MSDINATRLP	**IWGIGCNP**	CVGDEVAALLTRGEALC*	α-amanitin
	Ap16	MSDINATRLP	**IWGIGCNP**	CVGDEVTALITRGEALC*	α-amanitin
	Ap17	MSDINATRLP	**LGRPESLP**	CVGDDVNYILVSGGNLS*	
	Ap18	MSDINAARLP	**LVYMILFP**	SVGDDIDVVLGRGENLC*	
	Ap19	MSDVNATRLP	**MAFPEFLA**	CVGDDVNHTLTRGERLC*	
	Ap20	MSDINTARLP	**MHILAPPP**	CVSDDIEMVLTRGESLC*	
	Ap21	MSDINAARLP	**NLFVWIPP**	CISDDINSTLTRGESLC*	
	Ap22	MSDINTTRLP	**YMWDHHLP**	CASDDIQMVFTRGENLC*	
	Ap23	MSDINASRLP	**AWLATCP**	CAGDDVNPTLTRGESLC*	phalloidin
	Ap24	MSDINATRLP	**AWLMTCP**	CVGDDVNPTLTRGESLC*	“phallotoxin”
	Ap25	MSDVNATRLP	**AWLVDCP**	CVGDDINRLLTRGENLC*	phallacidin
*A. rimosa*	Ar1	MSDINTSRLP	**FIPLGIITILP**	CVSDDVNTTITRGESLC*	
	Ar2	MTDINDTRLP	**FVWILWLWLA**	CVGDDTSILNRGEDLC*	
	Ar3	MSDINATRLP	**IIIVLGLIIP**	LCVSDIEMILTRGESLC*	
	Ar4	MSDVNTTRLP	**FNFFRFPYP**	CICDDSEKVLELGENLC*	
	Ar5	MSDINATRLP	**HPFPLGLQP**	CAGDVDNFTVSCHSLC*	
	Ar6	MLDINATRFP	**LGRPTHLP**	CVGDDVNYILIGNGENLC*	
	Ar7	MSDINASCLP	**LILVANGMA**	YVSDDVSPTLTRGENLC*	
	Ar8	MPDINVTRLP	**LLIIVLLTP**	CISDDNNILNRGKDLC*	
	Ar9	MSDIHAARLP	**FPTRPVFP**	SAGDDMIEVVLGRGEDLC*	
	Ar10	MSDNNAARLP	**FYFYLGIP**	SDDAHPILTRGESLC*	
	Ar11	MSDINIARLP	**IFWFIYFP**	CVGDDVDNTLSRGESLS*	
	Ar12	MSDINASRLP	**ILKKPWAP**	SVCDDVNSTLTRGEGLC*	
	Ar13	MSDINVARLP	**ISDPTAYP**	CVGDDIQAVVKRGESLC*	
	Ar14	MSDINATRLP	**IWGIGCDP**	CVGDDVAALTTRGEALC*	β-amanitin
	Ar15	MSDINSTRLP	**IWGIGCNP**	SVGDEVTALLTRGEALC*	α-amanitin
	Ar16	MSDINATRLP	**AWDSKHP**	CVGDDVSRLLTRGESLC*	
	Ar17	MSDINATRVP	**AWLAECP**	CVGDDISHLLTRGENLC*	“phallotoxin”
	Ar18	MSDINATRVP	**AWLVDCP**	CVGDDISRLLTRGENLC*	phallacidin
*A. subpallidorosea*	Asp1	MTDVNDTRLP	**FIWLIWLWLP**	SVGDDINILNGGEDLC*	
	Asp2	MTDINYARLP	**ITLFLFFFIP**	CLSDDDNILNRGKDLC*	
	Asp3	MSDINTARLP	**YFLPPIFSPP**	CVSDDIEMVLTRGENLC*	
	Asp4	MSDINATRLP	**HPFPLGLQP**	CAGDVDNFTLTKGEDLC*	
	Asp5	MSDINATRLP	**GILIVWPP**	CVGDDVNFTLTRGESLC*	
	Asp6	MSDINTTRLP	**IAFPEFIA**	RVGDDIHRTLTRGESLC*	
	Asp7	MSDINVTRLP	**IFWFIYFP**	CVGDDVDNTLTRGESLS*	
	Asp8	MSDINAIRLP	**IGRPENKP**	CVGGDVNYILISGEKLC*	
	Asp9	MSDINATRLP	**IVFLEFYS**	CVGDDVNSTLTRGESLC*	
	Asp10	MSDINATRLP	**IWGIGCDP**	CVGDDVAAFLTRGEALC*	β-amanitin
	Asp11	MSDINATRLP	**IWGIGCNP**	SVGDEVTALLTRGEALC*	α-amanitin
	Asp12	MSDINASRLP	**VIGLFGLP**	YVSDDVQPILTRGDSLC*	
	Asp13	MSDINASRLP	**VIPFLLPP**	CVSDDVNFTLTRGESLC*	
	Asp14	MSDINATRLP	**YFRPAPPP**	CVSDDINPILTCGESLC*	
	Asp15	MSDINAARLP	**AWITDCP**	CVGDDINRILTRGENIC*	“phallotoxin”
	Asp16	MSDINASRFP	**AWLATCP**	CVGDDVNPTIARGESLC*	phalloidin
	Asp17	MSDINATRLP	**AWLVTCP**	CVGDDVNFTLTRGESLC*	“phallotoxin”
	Asp18	MSDINATRLP	**AWLVTCP**	CVGDDVNPTITRGESLC*	“phallotoxin”
	Asp19	MSDINTIRIP	**GPFGFA**	YVGDEVENLLKRGESLS*	
*L. venenata*	Lv1	MDANATRLP	**IWGIGCNP**	WTPESVNDTLTKDLS*	α-amanitin
	Lv2	MDANSTRLP	**IWGIGCNP**	WAPESVNDTLTRGKDLC*	α-amanitin

The MSDIN members with underlined numbers were verified at the genomic level. “Phallotoxin” means a novel heptapeptide similar to the phallotoxin cyclopeptide and capable of containing tryptathione (Trp-Cys)

**Table 3:** MSDIN family members cloned from genomic DNA of twelve amanitin-producing mushrooms

Name	No.	Leader peptide	Core peptide	Recognition sequence	Product	GenBank accession no.
*A. exitialis*	Ae1^**a**^	MSDINATRLP	**FIWVFGIP**	GDIGTVLTRGENLC*		MN318165
	Ae2^**a**^	MSDINATRLP	**IIWIIGNP**	CVSDDVERILTRGESLC*		MN318166
	Ae3^**ab**^	MSDINATRLP	**IWGIGCDP**	CVGDDVTALLTRGEALC*	β-amanitin	MN264225
	Ae4^**ab**^	MSDINATRLP	**IWGIGCNP**	CVGDDVTSVLTRGEALC*	α-amanitin	MN264220
	Ae5^**b**^	MSDINATRLP	**AWLTDCP**	CVGDDVNRLLTRGESLC*	“phallotoxin”	MN264235
	Ae6^**b**^	MSDINATRLP	**AWLVDCP**	CVGDDVNRLLTRGESLC*	phallacidin	MN264231
	Ae7^**a**^	MSDINATRLP	**VWIGYSP**	CVGDDCIALLTRGEGLC*		MN318167
	Ae8^**a**^	MSDINATRLP	**GFLFWA**	YVGDDVDYILTRGESLA*		MN318168
	Ae9^**a**^	MSDINATRLP	**GFLLWA**	YVGDDVDYILTRGESLA*		MN318169
*A. fuliginea*	Af1^**a**^	MSDINATRLP	**FPHFPPYNPP**	CVSDDIHMVLTRGENLC*		MN318170
	Af2^**a**^	MSDINATRLP	**YYLLLILPP**	CVSDDLQTVLTRGENLC*		MN318171
	Af3^**a**^	MSDINATRLP	**IFWFIYFP**	CVGDDVDNTLARGESLS*		MN318172
	Af4^**b**^	MSDINATRLP	**IWGIGCDP**	CVGDDVAALITRGEALC*	β-amanitin	MN264226
	Af5^**a**^	MSDINATRLP	**IWGIGCDP**	CVGEDVAALITRGEALC*	β-amanitin	MN318173
	Af6^**a**^	MSDINATRLP	**IWGIGCNP**	SVGDEVTALLTSGEALC*	α-amanitin	MN318174
	Af7^**a**^	MSDINATRLP	**LPSRPVFP**	FVSDAIEVVLGRGEDLC*		MN318175
	Af8^**b**^	MSDINASRLP	**AWLATCP**	CIGDDVNPTITRGESLC*	phalloidin	MN264249
	Af9^**ab**^	MSDINATRLP	**AWLVDCP**	CVGDDVNRLLARGENLC*	phallacidin	MN264232
*A. molliuscula*	Am1^**b**^	MSDINATRLA	**IWGIGCDP**	CVGDDVTALLTRGEALC*	β-amanitin	MN264227
*A. pallidorosea*	Ap1^**a**^	MSDINATRLP	**LIFIPPFIPP**	CVSDDIQMVLTRGENLC*		MN318176
	Ap2^**a**^	MSDINAPRLP	**LIFIPPFIPP**	CVSDDIQMVLTRGEGLC*		MN318177
	Ap3^**a**^	MSDINATRLP	**IPFHIPAP**	SVGDDIEVVLGRGENLC*		MN318178
	Ap4^**a**^	MSDINATRLP	**IWGIGCDP**	CVGDDVTAVLTCGEALC*	β-amanitin	MN318179
	Ap5^**b**^	MSDINATRLP	**IWGIGCDP**	CVGDDVTAVLTRGEALC*	β-amanitin	MN264228
	Ap6^**ab**^	MSDINATRLP	**IWGIGCNP**	CVGDEVAALLTRGEALC*	α-amanitin	MN264222
	Ap7^**b**^	MSDINATRLP	**IWGIGCNP**	CVGDEVTALITRGEALC*	α-amanitin	MN264221
	Ap8^**a**^	MSDINATRLP	**AWLATCP**	CAGDDVNPTLTRGESLC*	phalloidin	MN318180
	Ap9^**a**^	MSDINATRLP	**AWLMTCP**	CVGDDVNPILTRGESVC*	“phallotoxin”	MN318181
	Ap10^**b**^	MSDINATRLP	**AWLMTCP**	CVGDDVNPTLTRGESLC*	“phallotoxin”	MN264236
	Ap11^**ab**^	MSDVNATRLP	**AWLVDCP**	CVGDDINRLLTRGENLC*	phallacidin	MN264233
*A. rimosa*	Ar1^**a**^	MSDINATRLP	**IWGIGCDP**	CVGDDVAALATRGEALC*	β-amanitin	MN318182
	Ar2^**ab**^	MSDINATRLP	**IWGIGCDP**	CVGDDVAALTTRGEALC*	β-amanitin	MN264229
	Ar3^**a**^	MSDINATRLP	**IWGIGCNP**	SVGDEVTALLASGEALC*	α-amanitin	MN318183
	Ar4^**ab**^	MSDINSTRLP	**IWGIGCNP**	SVGDEVTALLTRGEALC*	α-amanitin	MN264223
	Ar5^**b**^	MSDINATRVP	**AWLAECP**	CVGDDISHLLTRGENLC*	“phallotoxin”	MN264237
*A. subfuliginea*	Asf1^**a**^	MSDINATRLP	**HPFPLGLQP**	CAGDVDNFTLTKGEGLC*		MN318184
	Asf2^**a**^	MSDINATRLP	**AIFLAWPP**	CVGDNVNSTLTRGESLC*		MN318185
	Asf3^**a**^	MSDINATRLP	**IWGIGCDP**	CVSDDVAALLTRGEALC*	β-amanitin	MN318186
	Asf4^**a**^	MSDINATRLP	**IWGIGCNP**	CVGDEVAALLTRGEALC*	α-amanitin	MN318187
	Asf5^**a**^	MSDINATRLP	**AWLVDCP**	CVGDDVNRLITRGENLC*	phallacidin	MN318188
*A. subjunquillea*	Asj1^**a**^	MSDINATRLP	**AYLPLFFIPP**	CVSDDIEMVLTRGESLC*		MN318189
	Asj2^**a**^	MSDINATRLP	**AYLPLFFIPP**	CVSDDIEVVLTRGESLC*		MN318190
	Asj3^**a**^	MSDINATRLP	**IWGIGCDP**	CIGDDVTALLTRGEALC*	β-amanitin	MH142177
	Asj4^**a**^	MSDINATRLP	**IWGIGCDP**	CVGDEVTALLTRGEALC*	β-amanitin	MH142176
	Asj5^**a**^	MSDINATRLP	**IWGIGCNP**	CVGDEVAALLTRGEALC*	α-amanitin	MH142175
	Asj6^**ab**^	MSDINATRLP	**AWLATCP**	CAGDDVNPTLTRGESLC*	phalloidin	MN264250
	Asj7^**a**^	MSDINATRLP	**AWLATCP**	CVGDDVNPTLSRGESLC*	phalloidin	MN318191
	Asj8^**ab**^	MSDINATRLP	**AWLVDCP**	CVGDDINRLLTRGENLC*	phallacidin	MN264234
*A. subpallidorosea*	Asp1^**ab**^	MSDINATRLP	**IWGIGCDP**	CVGDDVAAFLTRGEALC*	β-amanitin	MN264230
	Asp2^**ab**^	MSDINATRLP	**IWGIGCNP**	SVGDEVTALLTRGEALC*	α-amanitin	MN264224
	Asp3^**ab**^	MSDINAARLP	**AWITDCP**	CVGDDINRILTRGENIC*	“phallotoxin”	MN272408
	Asp4^**ab**^	MSDINASRFP	**AWLATCP**	CVGDDVNPTIARGESLC*	phalloidin	MN272407
	Asp5^**a**^	MSDINATRLP	**AWLITCP**	CVGDDANPTITRGESLC*	“phallotoxin”	MN318192
	Asp6^**ab**^	MSDINATRLP	**AWLVTCP**	CVGDDVNPTITRGESLC*	“phallotoxin”	MN272409
	Asp7^**a**^	MSDINATRLP	**AWLVTCP**	CVGDDVNSTITRGESLC*	“phallotoxin”	MN318193
*A. virosa*	Av1^**a**^	MSDINATRLP	**FLLFIIPP**	CVSDDVNSTLTRGESLC*		MN318194
	Av2^**a**^	MSDINATRLP	**FYFQPGFP**	WSVGDDVNPTLTRGESLC*		MN318195
	Av3^**b**^	MSDINATRLP	**IWGIGCNP**	SVGDEATALLTRGEALC*	α-amanitin	MN272412
	Av4^**a**^	MSDINATRLP	**SILIVWPP**	CVGDDVNSTLTRGESLC*		MN318196
	Av5^**a**^	MSDINATRLP	**SILVVWPP**	CVSDDVNSTLTRGESLC*		MN318197
	Av6^**a**^	MSDINATRLP	**AWLATCP**	CVGDDVNPTLARGESLC*	phalloidin	MN318198
	Av7^**a**^	MSDINATRLP	**AWLVDCP**	CVGDDINRLLTRGENLC*	phallacidin	MN318199
	Av8^**a**^	MSDINATRLP	**AWLVTCP**	CVGDDVNPTLTRGESLC*	“phallotoxin”	MN318200
	Av9^**a**^	MSDINATRLP	**GPFLFFP**	FVSDDIEVILRRGEDLC*		MN318201
*G. marginata*	Gm1^**b**^	MFDTNATRLP	**IWGIGCNP**	WTAEHVDQTLASGNDIC*	α-amanitin	MN272413
	Gm2^**b**^	MFDTNSTRLP	**IWGIGCNP**	WTAEHVDQTLVSGNDIC*	α-amanitin	MN272414
*G. sulciceps*	Gs1^**b**^	MFDTNATRLP	**IWGIGCNP**	WTAEHVDQTLASGNDIC*	α-amanitin	MN272417
	Gs2^**b**^	MFDTNSTRLP	I*GIGCNP	WTAEHIDQTLVSGNDTC*		MN272418
*L. venenata*	Lv1^**b**^	MDANATRLP	**IWGIGCNP**	WTPESVNDTLTKDLS	α-amanitin	MN272421
	Lv2^**b**^	MDANSTRLP	**IWGIGCNP**	WAPESVNDTLTRGKDLC	α-amanitin	MN272422

Superscripts a and b are for products cloned with degenerate and specific primers, respectively. “Phallotoxin” means a novel heptapeptide similar to phallotoxin cyclopeptide and capable of containing Tryptathione (Trp-Cys)

Among the MSDIN members found in the 9 lethal species of *Amanita* sect. *Phalloideae* included in our study, in addition to the common α-amanitin, β-amanitin, phallacidin and phalloidin (PHA) peptides, several unnamed predicted peptides overlapped among different *Amanita* species, including “FNFFRFPYP” in *A. exitialis* and *A. rimosa*; “FPWTGPFVP” in *A. fuliginea* and *A. pallidorosea*; “IIIVLGLIIP” in *A. fuliginea* and *A. rimosa*; “YFLPPIFSPP” in *A. molliuscula* and *A. subpallidorosea*; “ISDPTAYP” in *A. pallidorosea* and *A. rimosa*; “IFWFIYFP” in *A. exitialis*, *A. fuliginea*, *A. rimosa* and *A. subpallidorosea*; and “ISDPTAYP” in *A. pallidorosea*, *A. rimosa*, *A. subfuliginea* and *A. subpallidorosea*. The remaining 87 core regions were unique to their corresponding species. The MSDIN genes encoding “AWLTDCP” in *A. exitialis*; “AWLMTCP” in *A. pallidorosea*; “AWLECP” in *A. rimosa*; “AWLVTCP” in *A. fuliginea*, *A. subpallidorosea* and *A. virosa*; and “AWITDCP” and “AWLITCP” in *A. subpallidorosea* probably produce new unknown phallotoxins because their core regions are similar to those of phallacidin (AWLVDCP) and phalloidin (AWLATCP). As expected, no MSDIN genes were found in *A. oberwinklerana*, a species belonging to *Amanita* sect. *Lepidella* [16] or sect. *Roanokenses* that dose not contain cyclopeptide toxins [15].

In *G. marginata*, *G. sulciceps* and *L. venenata*, only MSDIN genes encoding α-amanitin were found, and such genes were the only genes common to the amanitin-producing genera *Amanita*, *Galerina* and *Lepiota*. Unlike the situation in lethal *Amanita* species, no MSDIN genes other than the α-amanitin gene were discovered. Interestingly, an MSDIN gene with the full amino acid sequence MFDTNSTRLPI*GIGCNPWTAEHIDQTLVSGNDTC* (with the core region shown in bold and underlined) was found in *G. sulciceps*. Due to its similarity to the α-amanitin gene *Gs_α*-*AMA1* (MFDTNATRLP**IWGIGCNP**WTAEHVDQTLASGNDIC*) in *G. sulciceps*, it was designated *Gs_α*-*AMA2.*

Similarly, 19 *POP* genes were identified from the transcriptomes of nine *Amanita*, two *Galerina* and one *Lepiota* species using known *POPA* and *POPB* genes of *A. bisporigera* and *G. marginata*, respectively, as the TBLASTN queries and these sequences were further verified by PCR amplification. Eleven lethal *Amanita*, *Galerina* and *Lepiota* species contained both *POPA* and *POPB* genes, but *A. oberwinklerana*, an *Amanita* species producing no cyclopeptide toxins, only exhibited the *POPA* gene. All of the obtained *POP* sequences and their accession numbers are listed in Table 4.

**Table 4:** Gene sequences used in the molecular phylogenetic analyses and their GenBank accession numbers

Taxon	Gene	Source	GenBank accession no.
*Agaricus bisporus* var. *bisporus*	*POP*	NCBI	XM006459721
*Agaricus bisporus var. burnettii*	*POP*	NCBI	JH971409
*Amanita bisporigera*	*α-AMA1*	Pulman et al., 2016	–
	*α-AMA2*	Pulman et al., 2016	–
	*PHA1*	Pulman et al., 2016	–
	*PHA2*	Pulman et al., 2016	–
	*POPA*	Pulman et al., 2016	–
	*POPB*	Pulman et al., 2016	–
*Amanita exitialis*	*α-AMA*	Our study	MN264220
	*β-AMA*	Our study	MN264225
	*PHA*	Our study	MN264231
	“*AWLTDCP*”	Our study	MN264235
	*POPA*	Our study	MN264238
	*POPB*	Our study	MN264244
*Amanita fuliginea*	*β*-*AMA*	Our study	MN264226
	*PHA*	Our study	MN264232
	*PHD*	Our study	MN264249
	*POPA*	Our study	MN264239
	*POPB*	Our study	MN264245
*Amanita molliuscula*	*β-AMA*	Our study	MN264227
	*POPA*	Our study	MN264240
	*POPB*	Our study	MN264246
*Amanita muscaria*	*POPA*	NCBI	KN818232
*Amanita oberwinklerana*	*POPA*	Our study	MN264241
*Amanita pallidorosea*	*α*-*AMA1*	Our study	MN264221
	*α*-*AMA2*	Our study	MN264222
	*β-AMA*	Our study	MN264228
	*PHA*	Our study	MN264233
	“*AWLMTCP*”	Our study	MN264236
	*POPA*	Our study	MN264242
	*POPB*	Our study	MN264247
*Amanita phalloides*	*α*-*AMA*	Pulman. et al., 2016	–
	*β-AMA1*	Pulman. et al., 2016	–
	*β-AMA2*	Pulman et al., 2016	–
	*PHA*	Pulman et al., 2016	–
	*PHD1*	Pulman et al., 2016	–
	*PHD2*	Pulman et al., 2016	–
	*PHD3*	Pulman et al., 2016	–
	*POPA*	Pulman et al., 2016	–
	*POPB*	Pulman et al., 2016	–
*Amanita rimosa*	*α*-*AMA*	Our study	MN264223
	*β-AMA*	Our study	MN264229
	“*AWLAECP*”	Our study	MN264237
	*POPA*	Our study	MN264243
	*POPB*	Our study	MN264248
*Amanita subjunquillea*	*α*-*AMA*	Luo et al., 2018	–
	*β-AMA1*	Luo et al., 2018	–
	*β-AMA2*	Luo et al., 2018	–
	*PHA*	Our study	MN264234
	*PHD*	Our study	MN264250
	*POPA*	Luo et al., 2018	–
	*POPB*	Luo et al., 2018	–
*Amanita subpallidorosea*	*α*-*AMA*	Our study	MN264224
	*β-AMA*	Our study	MN264230
	*PHD*	Our study	MN272407
	“*AWITDCP*”	Our study	MN272408
	“*AWLVTCP*”	Our study	MN272409
	*POPA*	Our study	MN272410
	*POPB*	Our study	MN272411
*Amanita thiersii*	*POPA*	NCBI	KZ302001
*Amanita virosa*	*α*-*AMA*	Our study	MN272412
*Anomoporia bombycina*	*POP*	JGI	–
*Auriculariopsis ampla*	*POP*	NCBI	VDMD01000002
*Bolbitius vitellinus*	*POP*	JGI	–
*Conocybe apala*	*POP*	NCBI	FJ906819
*Coprinellus micaceus*	*POP*	NCBI	QPFP01000027
*Coprinopsis cinerea*	*POP*	NCBI	XM001841192
*Coprinopsis marcescibilis*	*POP*	NCBI	ML210154
*Cortinarius glaucopus*	*POP*	JGI	–
*Crucibulum laeve*	*POP*	NCBI	ML213591
*Cyathus striatus*	*POP*	JGI	–
*Fistulina hepatica*	*POP*	NCBI	KN881639
*Galerina marginata*	*α*-*AMA1*	Our study	MN272413
	*α*-*AMA2*	Our study	MN272414
	*POPA*	Our study	MN272415
	*POPB*	Our study	MN272416
*Galerina sulciceps*	*α*-*AMA1*	Our study	MN272417
	“*α*-*AMA2*”	Our study	MN272418
	*POPA*	Our study	MN272419
	*POPB*	Our study	MN272420
*Gymnopilus chrysopellus*	*POP*	JGI	–
*Gymnopilus dilepis*	*POP*	NCBI	NHYE01005597
*Hebeloma cylindrosporum*	*POP*	NCBI	KN831777
*Hypholoma sublateritium*	*POP*	NCBI	KN817688
*Hypsizygus marmoreus*	*POP*	NCBI	LUEZ02000233
*Laccaria amethystina*	*POP*	NCBI	KN838546
*Laccaria bicolor*	*POP*	NCBI	DS547115
*Lepiota brunneoincarnata*	*POPA*	NCBI	MN912699
*Lepiota subincarnata*	*α*-*AMA1*	Luo et al., 2018	–
	*α*-*AMA2*	Luo et al., 2018	–
	*POPB*	Luo et al., 2018	–
*Lepiota venenata*	*α*-*AMA1*	Our study	MN272421
	*α*-*AMA2*	Our study	MN272422
	*POPA*	Our study	MN272423
	*POPB*	Our study	MN272424
*Lepista nuda*	*POP*	JGI	–
*Leucoagaricus* sp.	*POP*	NCBI	KQ962668
*Macrolepiota fuliginosa*	*POP*	JGI	–
*Panaeolus cyanescens*	*POP*	NCBI	NHTK01005903
*Pleurotus ostreatus*	*POP*	NCBI	KL198007
*Plicaturopsis crispa*	*POP*	JGI	–
*Pterula gracilis*	*POP*	NCBI	ML178816
*Schizophyllum commune*	*POP*	NCBI	GL377318
*Termitomyces* sp.	*POP*	NCBI	KQ412502

### Comparison of MSDIN precursor peptide sequences

**Fig. 1:**
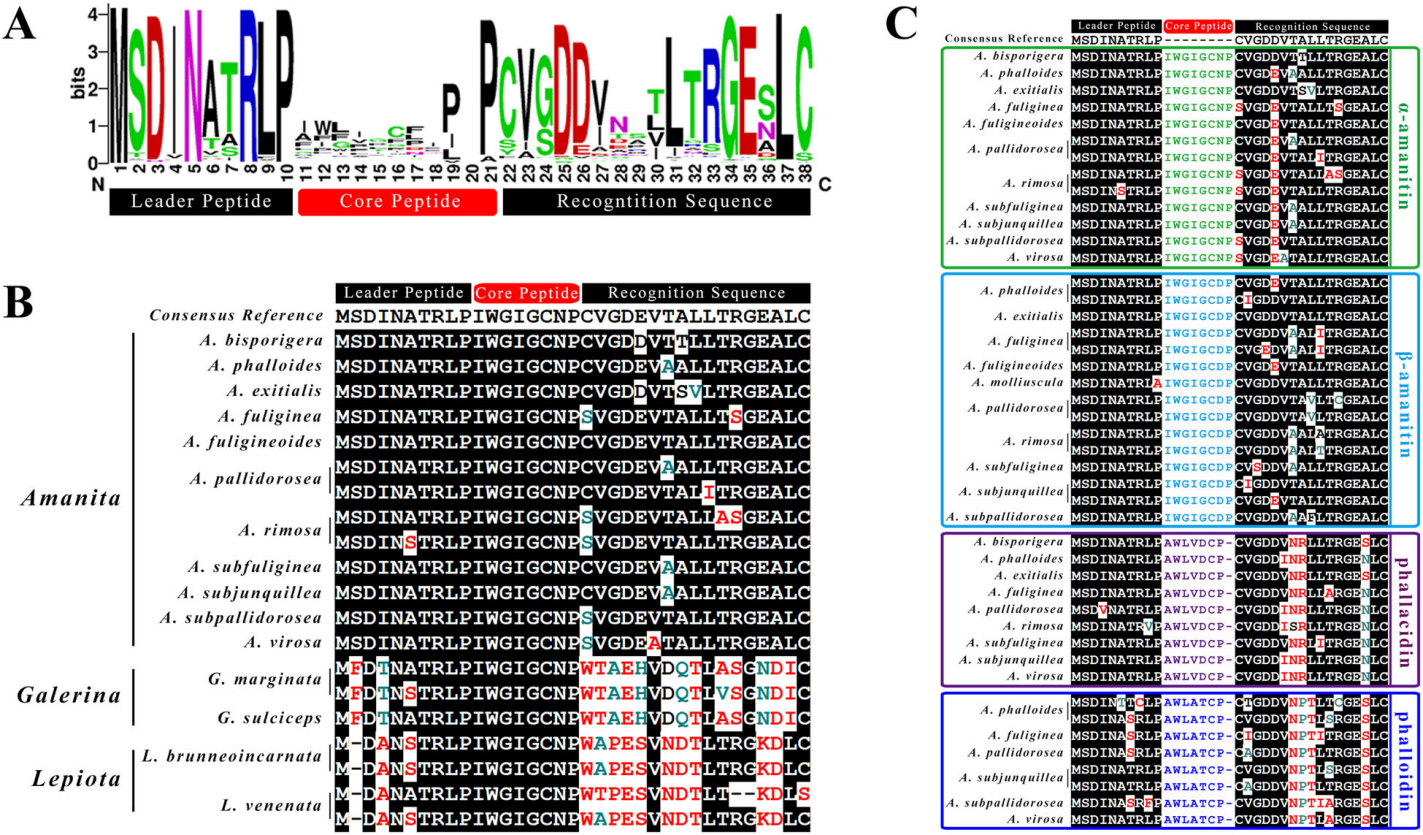
Alignment of MSDIN precursor peptide sequences. **a** WebLogo [27] alignment of 145 MSDIN members from 9 *Amanita* species. The letter height of each amino acid represents its conservation degree, and the higher the letter, the more conserved the site. **b** Alignment of the precursor peptide sequences of α-amanitin from 15 amanitin-producing mushrooms. **c** Alignment of the precursor peptide sequences of α-, β-amanitin, phallacidin and phalloidin from 12 *Amanita* species. Letters with white background are variations compared with the consensus sequence. The sequences of *A. bisporigera*, *A. phalloides* were from Pulman et al. (2016), the sequences of *A. fuligineoides* were from Li et al. (2014) and the sequences of *L. brunneoincarnata* were from Lüli et al. (2019)

WebLogo alignment was carried out for 145 MSDIN sequences obtained from 9 *Amanita* species (Fig. 1a). The composition and structure of these sequences and the relative degree of conservation of the amino acids at each point were analysed. As shown in Fig. 1a, the MSDIN precursor peptides of the *Amanita* species were 31–38 amino acids in length and could be divided into three regions: a highly conserved upstream leader peptide, a relatively conserved downstream recognition sequence and a highly variable core peptide. The core peptide was located between P^10^ and P^21^ and included the latter proline, and its ends were the leader peptide and recognition sequence of MSDIN. The leader peptide contained 10 amino acids, and the M^1^S^2^D^3^I^4^N^5^R^8^L^9^P^10^ residues were highly conserved, with conservation rates of 100% (145/145), 91.7% (133/145), 97.2% (141/145), 93.1% (135/145), 99.3% (144/145), 99.3% (144/145), 94.5% (137/145) and 99.3% (144/145), respectively. In the leader peptide, P^10^ was the first cleavage site for prolyl oligopeptidase (POPB) [12]. The recognition sequence usually contained 17 amino acids, beginning with C^22^V^23^G^24^D^25^D^26^, with conservation rates of 76.5% (111/145), 79.3% (115/145), 66.2% (96/145), 93.1% (135/145) and 83.4% (121/145), and ending with L^31^T^32^R^33^G^34^E^35^L^37^C^38^, with conservation rates of 86.9% (126/145), 73.8% (107/145), 82.1% (119/145), 96.6% (140/145), 93.1% (135/145), 97.9% (142/145) and 91.7% (133/145), respectively. In the recognition sequence, L^31^ and L^37^ were conducive to the formation of an alpha helix and substrate recognition by the POPB enzyme [28]; additionally, the C-terminal Cys^38^ (sometimes replaced with Ser) was indispensable for performing the function of POPB [12]. The core peptides were predicted to form cyclopeptides in which the last amino acid, P^21^ (the second cleavage site for POPB), was highly conserved, with a conservation rate of 94.5% [12].

The *α-AMA* precursor peptide sequences of the genera *Amanita*, *Galerina* and *Lepiota* were compared, as shown in Fig. 1b. The *α-AMA* sequences showed few differences within the same genus but presented more differences between the different genera. The *α-AMA* leader peptides of the three genera showed few differences and were more conserved than the other sequences. The leader peptides of *Amanita* and *Galerina* contained 10 amino acids, while that of the genus *Lepiota* contained 9, and the sequences of the three genera started with “MSDIN”, “MFDTN”, and “MDAN”, respectively. In the recognition sequences of the three genera, with the exception of several highly conserved amino acids (specifically V, L, G, and LC or LS at the end), many differences were observed. Overall, there were large differences among the *α-AMA* sequences of *Amanita*, *Galerina* and *Lepiota*, but the *Galerina* and *Lepiota α-AMA* sequences were closer to each other than to those of *Amanita*.

The *Amanita* MSDIN genes encoding amatoxins (α-, β-amanitin) and phallotoxins (phallacidin and phalloidin), which are the major cyclopeptides in these mushrooms, were aligned, and the highlighted variations were compared to representative consensus sequences (Fig. 1c). In general, the precursor peptide sequences encoding the same toxin shared high identity. There were obviously more variations in the recognition sequences than in the leader peptides. The phallotoxin sequences presented more variations than the amatoxin sequences.

### Structures of MSDIN and *POP* genes

**Fig. 2:**
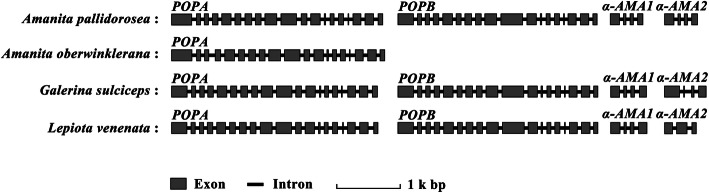
Structures of *α-AMA* and *POP* exemplified by four agaric species

The genomic sequences and coding sequences of the toxin MSDIN and *POP* genes obtained in this study (Table 2)were subjected to pairwise alignment, and the gene composition of the exons and introns was analysed. As shown in Fig. 2, the *POPA* genes comprised 19 exons and 18 introns, while the *POPB* genes comprised 18 exons and 17 introns, which was very similar to other known *POP* genes. The *α-AMA* genes of *Amanita* and *Galerina* contained three introns, while the *α-AMA* genes of *Lepiota* contained two or three introns. In addition to *α-AMA*, other MSDIN toxin genes in *Amanita* species, such as *β-AMA*, *PHA* and *PHD*, were also composed of three introns.

### Phylogenetic analysis of MSDIN and *POP* genes

**Fig. 3:**
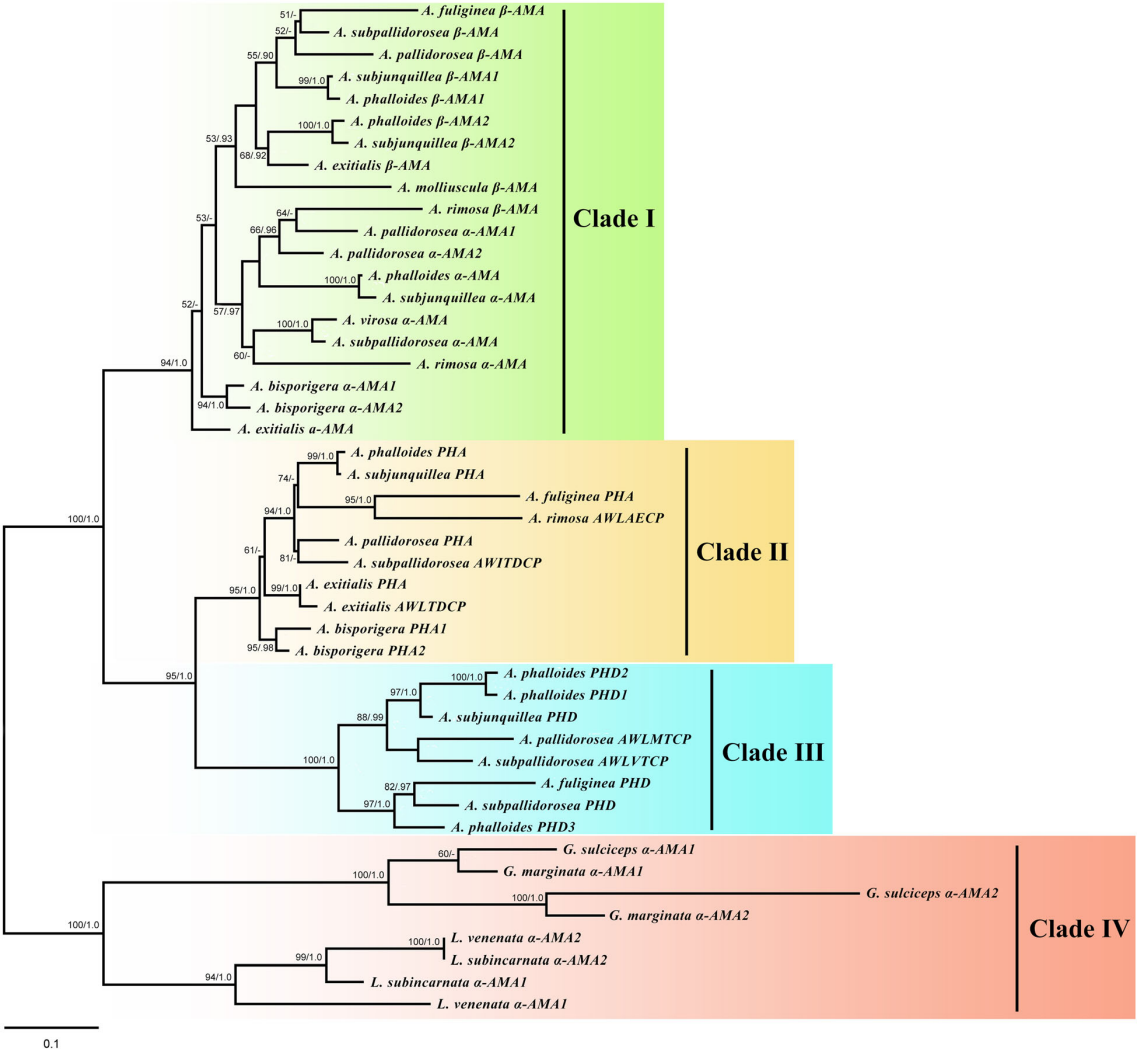
Phylogenetic trees generated from maximum likelihood analysis based on toxin MSDIN genes. Bootstrap percentages (> 50%) based on 1000 replications and Bayesian posterior probabilities (> 0.90) are shown at nodes. Bar, a substitution per 10 nucleotides

**Fig. 4:**
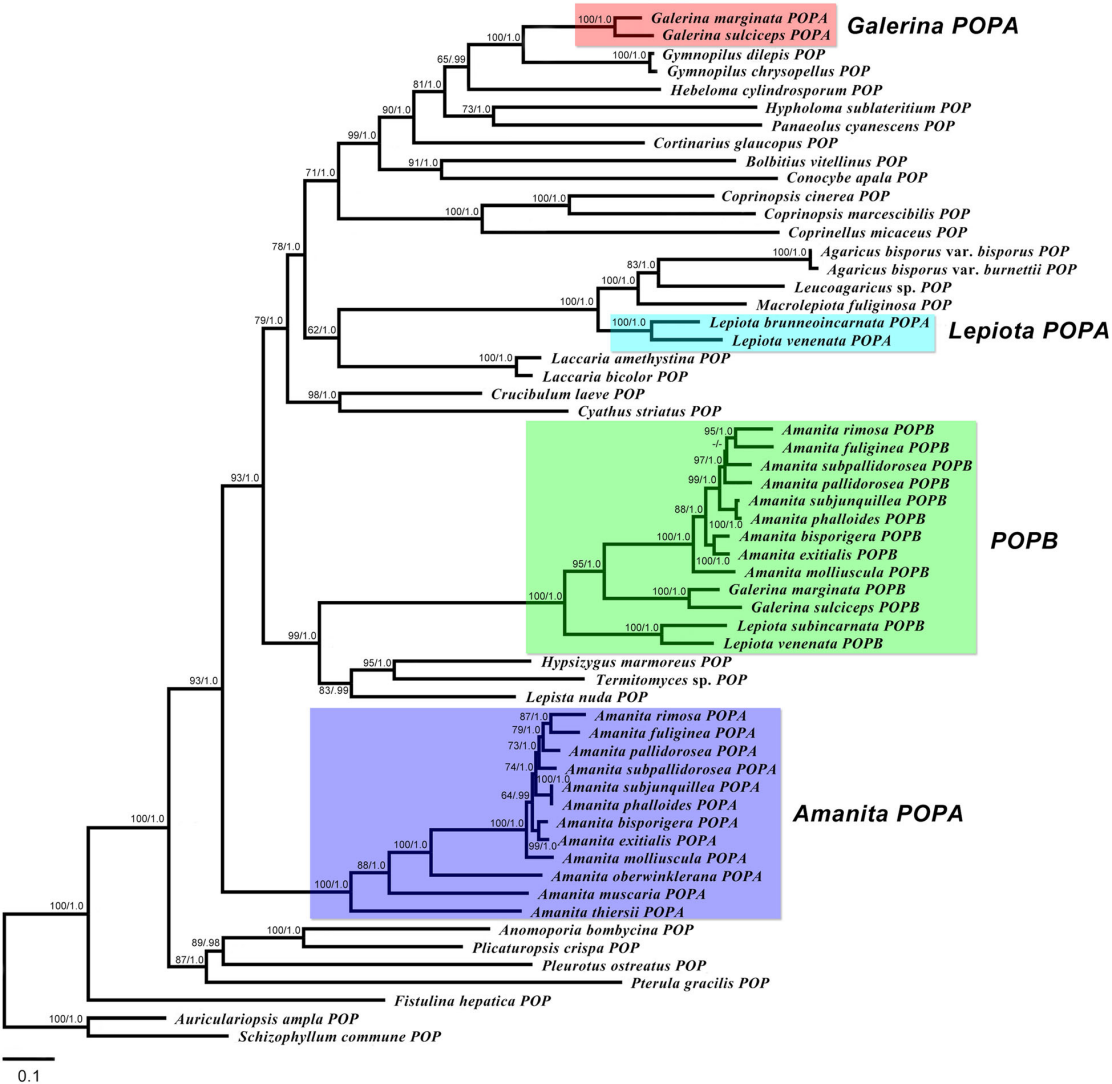
Phylogenetic trees generated from maximum likelihood analysis based on *POP* genes. Bootstrap percentages (> 50%) based on 1000 replications and Bayesian posterior probabilities (> 0.90) are shown at nodes. Bar, a substitution per 10 nucleotides

From the phylogenetic analysis, two maximum likelihood (ML) trees based on 46 MSDIN toxin genes from 14 amanitin-producing mushrooms and 58 *POP* genes from 46 agaric species were constructed. In the MSDIN toxin gene tree (Fig. 3), all MSDIN toxin gene sequences were distributed in four clades. Clade I contained 10 α-amanitin gene sequences and 10 β-amanitin gene sequences from 10 lethal *Amanita* species forming a cluster with 95% bootstrap support and a Bayesian posterior probability of 1.0. Clade II contained MSDIN genes encoding AWLVDCP (phallacidin, PHA) and the unknown related variants AWLAECP, AWITDCP and AWLTDCP forming a cluster with 95% bootstrap support and a Bayesian posterior probability of 1.0. Clade III contained MSDIN genes encoding AWLATCP (phalloidin, PHD) and the unknown related variants AWLMTCP and AWLVTCP forming a cluster with a 100% bootstrap and a 1.0 Bayesian posterior probabilities. Clade IV contained α-amanitin genes from *Galerina* and *Lepiota* species, including *G. marginata*, *G. sulciceps*, *L. subincarnata* and *L. venenata*, forming a cluster with a 100% bootstrap and a 1.0 Bayesian posterior probabilities. In the *POP* gene tree (Fig. 4), *POPA* sequences from *Amanita*, *Galerina* and *Lepiota* were separated from each other in different groups. *Amanita POPA* sequences (12) were clustered together as a single group, while *Galerina POPA* sequences (2) were clustered in a group containing *Gymnopilus dilepis* and *Gymnopilus chrysopellus*, and *Lepiota POPA* sequences (2) were clustered in a group containing *Agaricus bisporus* var. *bisporus*, *Agaricus bisporus* var. *burnettii*, *Leucoagaricus* sp. and *Macrolepiota fuliginosa*. However, *POPB* sequences (13) belonging to three disjunct genera (*Amanita*, *Galerina* and *Lepiota*) were clustered together forming a monophyletic group.

## Discussion

It has been proven that lethal *Amanita* species are classified in section *Phalloideae* of *Amanita* and that these species contain members of the MSDIN gene family, allowing them to produce many small cyclopeptides, such as α-amanitin, on ribosomes [6, 16]. In the present study, nine lethal *Amanita* species from China, including *A. exitialis*, *A. fuliginea*, *A. mulliuscula*, *A. pallidorosea*, *A. rimosa*, *A. subfuliginea*, *A. subjunquillea*, *A. subpallidorosea* and *A. virosa*, were proven to contain MSDIN genes, as found in other lethal *Amanita* species described previously, such as *A. bisporigera*. These results further suggest that species of the *Amanita* section *Phalloideae* are genetically similar and are able to biosynthesize amatoxins, phallotoxins and some other unknown peptides.

Based on the MSDIN gene data from nine lethal *Amanita* species from China obtained in our study and some other European and North American species, such as *A. bisporigera*, *A. phalloides* and *A. ocreata* [5, 6], most MSDIN genes have not been found to be common and may even be unique among these lethal *Amanita* species. In species of *Amanita* section *Phalloideae*, the MSDIN gene encoding α-amanitin is present in all species, whereas the MSDIN genes encoding β-amanitin, phallacidin and phalloidin are widely distributed but are not common to all species. This findings suggested that each lethal *Amanita* species exhibits its own independent MSDIN family and that few overlapping MSDIN genes occur among lethal *Amanita* species.

In addition to species of *Amanita* section *Phalloideae*, some *Galerina* and *Lepiota* species, such as *G. marginata* and *L. brunneoincarnata*, produce amatoxin [2, 29]. In our study, the MSDIN gene-mining results showed that *G. marginata* and *L. venenata* only presented two copies of the α-amanitin gene, and no additional MSDIN genes were found, consistent with the results of Luo et al. (2012) and Lüli et al. (2019) [7, 9]. Lethal *Galerina* and *Lepiota* species are considered to have two copies of the α-amanitin gene. However, the analysis of the MSDIN genes of another *Galerina* species, *G. sulciceps*, showed that *G. sulciceps* only presented a single copy of the α-amanitin gene, although it also exhibited an MSDIN gene that was extremely similar to the α-amanitin gene with an I*GIGCNP core region. This MSDIN gene seemed to represent an α-amanitin gene mutation, and we speculated that its tryptophan (W) codon, TGG, in the core region has been mutated to a termination codon, TGA, via a single-base substitution, thus inhibiting the proper expression of the gene. In general, only the α-amanitin gene is found in *Amanita*, *Galerina* and *Lepiota*, which indicates that the α-amanitin genes of the three genera might share a common origin or originate from a single genus. Additionally, MSDIN genes including *β-AMA*, *PHA*, *PHD*, etc., were only found in *Amanita*, which indicated that these MSDIN genes (except for *α-AMA*) were likely derived from lethal *Amanita* species. The higher genetic diversity of MSDIN genes in *Amanita* than in *Galernia* and *Lepiota* causes the lethal *Amanita* species to produce greater amounts of toxic compounds than *Galernia* and *Lepiota* species. For this reason, lethal *Amanita* species present a greater defence ability to prevent their consumption than *Galernia* and *Lepiota* species.

Lethal *Amanita* species contain three primary kinds of peptide toxins: amatoxins, phallotoxins and virotoxins [21]. MSDIN genes encoding amatoxin and phallotoxin were discovered in 2007 [5], but there has been no related evidence of MSDIN genes encoding virotoxins published to date. It has been reported that *A. subpallidorosea* and *A. virosa* contain virotoxins [3, 30]. In this study, toxin genes of the two lethal *Amanita* species were also identified, and no virotoxin genes were found. Nevertheless, the two species both contain MSDIN genes encoding AWLATCP (PHD) and AWLVTCP, which only show a single amino acid difference in the composition of the virotoxins (AWLATSP or AWLVTSP). Therefore, we speculated that virotoxins might be encoded by the *PHD* gene or the phallotoxin-like gene AWLVTCP and that cysteine (C) is transformed to serine (S) during posttranslational modification.

Phylogenetic analysis showed that the *Galerina α-AMA* genes and *Lepiota α-AMA* genes were homologous but were distant from the *Amanita α-AMA* gene. In the genus *Amanita*, *α-AMA* and *β-AMA* are mixed and clustered in a clade, which indicates that *β-AMA* might be derived from *α-AMA*. *PHA* genes (AWLVDCP) were clustered with MSDIN genes encoding AWLAECP, AWITDCP and AWLTDCP, and *PHD* genes (AWLATCP) were clustered with MSDIN genes encoding AWLMTCP and AWLVTCP, which indicated that the encoded products of these MSDIN genes were very likely to correspond to new unknown phallotoxins, considering the similarity of their amino acid composition with those of PHA and PHD and their capacity to contain tryptathione (Trp-Cys). These phallotoxin-like genes might be variants derived from *PHA* and *PHD*. For example, we found that the *PHA* gene (AWLVDCP) sequence in *A. exitialis* was almost the same as the sequence of the MSDIN gene encoding AWLTDCP, with only a two-nucleotide difference in the core region, and the valine (V) codon GTA is likely mutated into the threonine (T) codon ACA. According to this finding, it can be inferred that the MSDIN genes in *Amanita* evolved faster than those in *Galerina* and *Lepiota*, which led to the generation of a variety of new peptide genes and might also be the reason why the *Galerina* and *Lepiota α-AMA* genes differed from the *Amanita α-AMA* genes.

Horizontal gene transfer (HGT), also known as lateral gene transfer, refers to the transmission of genetic material between distinct organisms, specifically across species boundaries [31, 32]. It has been reported that HGT is very common in prokaryotes and may be an important source of their biological evolution, and HGT also occurs in eukaryotes at a lower frequency than in prokaryotes [33–36]. The most recent reports suggest that HGT may be responsible for the α-amanitin biosynthetic pathway found in the three distantly related genera *Amanita*, *Galerina* and *Lepiota* [8, 9]. It has been reported that in amanitin-producing mushrooms, the *POPB* gene product catalyses the cleavage and cyclization of the toxin precursor peptide, while the *POPA* gene is a housekeeping gene that is unrelated to toxin biosynthesis [7, 12]. In our study, phylogenetic analysis based on the *POP* gene showed that the *POPA* genes of *Amanita*, *Galerina* and *Lepiota* were distributed in three separate groups, but the *POPB* genes of the three genera were highly homologous forming a highly monophyletic group, which apparently conflicted with the species taxonomic status and could not be explained by conserved gene inheritance. Additionally, the MSDIN and *POP* genes were proven to exhibit the same exon and intron structures. These results can be considered to represent evidence of HGT events among *Amanita*, *Galerina* and *Lepiota*. For the complete validation of HGT among amanitin-producing mushrooms in the future, the inclusion more related species and their genomic data will be required to perform a phylogenetic analysis with appropriate taxon sampling and tree-building methodologies.

## Conclusions

In conclusion, the MSDIN gene family is abundant and diverse. In addition to the peptide toxins α-amanitin, β-amanitin, phallacidin, phalloidin, etc., the MSDIN family encodes a variety of unknown small cyclopeptides. The amanitin-producing species *Amanita*, *Galerina* and *Lepiota* exhibit a common toxin biosynthetic pathway, and their α-amanitin genes and *POPB* genes may have a common origin that involving HGT among the three distant genera.

## Methods

### Sample collection and preparation

Samples of seven *Amanita* species and *Lepiota venenata* were collected from the wild for RNA extraction and sequencing, and their fresh basidiocarps were cleaned and placed on dry ice then transported back to the lab and stored at − 80 °C. The mushroom samples intended for DNA extraction were dried with silica gel and then stored at 4 °C. The mycelia of two *Galerina* strains were cultivated to grow material for DNA and RNA extraction. Detailed information for the mushroom materials used in this study is given in Table 5.

**Table 5:** Information of the mushroom materials used in this study

Species name	Locality	Collection time	Specimen no.	GenBank accession no.	Nucleic acid extract
*A. exitialis*	Guangdong, China	2017-03-27	MHHNU 30937	KR996717	DNA, RNA
*A. fuliginea*	Hunan, China	2017-06-06	MHHNU 9047	MN061271	DNA, RNA
*A. molliuscula*	Jilin, China	2017-08-07	MHHNU 9142	MN061272	DNA, RNA
*A. oberwinklerana*	Hunan, China	2017-06-09	MHHNU 9051	MN061273	DNA, RNA
*A. pallidorosea*	Shandong, China	2018-08-13	MHHNU 31203	MN061274	DNA, RNA
*A. rimosa*	Hunan, China	2017-06-09	MHHNU 9050	MN061275	DNA, RNA
*A. subfuliginea*	Chongqing, China	2015-07-01	MHHNU 30946	MN061276	DNA
*A. subjunquillea*	Hunan, China	2012-09-10	MHHNU 7751	KR996715	DNA
*A. subpallidorosea*	Hunan, China	2017-09-14	MHHNU 8617	KU601411	DNA, RNA
*A. virosa*	Hunan, China	2016-09-09	MHHNU 8621	KY472227	DNA
*G. marginata*	–	–	MHHNU 8380	MN061277	DNA, RNA
*G. sulciceps*	–	–	MHHNU 7669	KX214585	DNA, RNA
*L. venenata*	Hubei, China	2017-9-10	MHHNU 31031	MK095189	DNA, RNA

*G. marginata* and *G. sulciceps* samples were cultured mycelia, and the other mushroom samples were wild fruiting bodies

### Nucleic acid extraction and cDNA preparation

Total genomic DNA was extracted using the Fungal DNA Mini Kit (Omega Bio-tek, Norcross, USA). Total RNA was isolated using TRIzol Reagent (Invitrogen, Carlsbad, USA) following the TRIzol User Guide. cDNA was synthesized using TransScript® One-Step gDNA Removal and cDNA Synthesis SuperMIX (Transgen Biotech, Beijing, China). The DNA and RNA quality and yield were detected using a SmartSpec Plus (Bio-Rad, Hercules, USA).

### Transcriptome sequencing and de novo assembly

The concentration, purity and integrity of the RNA samples used for next-generation sequencing were further examined using an Agilent 2100 bioanalyzer (Agilent, Santa Clara, USA). Qualified RNA samples were used to construct circular single-stranded cDNA libraries, and the libraries were then sequenced on a BGISEQ-500 sequencer (BGI, Shenzhen, China). Clean reads were obtained using the filtering software SOAPnuke to remove reads containing adaptors, reads with more than 5% unknown bases, and low-quality reads (bases with a quality value < 15 accounting for more than 20% of the bases in a read) from the raw reads. These clean reads were de novo assembled using Trinity software. Finally, nonredundant unigenes were obtained using Tgicl software. All of these steps were performed by the Beijing Genomic Institute (BGI)-Wuhan in China.

### Retrieval and annotation of MSDIN and POP genes

The unigene data obtained as described above were searched for MSDIN and *POP* genes (*Galerina* unigenes were provided by Professor Ping Zhang at Hunan Normal University) by using the known amino acid sequences of the MSDIN family and *POP* genes from *A. bisporigera* and *G. marginata* [5, 7] as queries for the online NCBI TBLASTN tool. Then, unigenes similar to the queries were manually annotated, and the coding sequences were predicted and translated into protein sequences using DNAMAN 7.0 software.

### Cloning of MSDIN and *POP* genes

Partial MSDIN gene sequences were amplified from *Amanita* genomic DNA using the following degenerate primers: forward (5′-ATGTCNGAYATYAAYGCNACNCG-3′) and reverse (5′-CCAAGCCTRAYAWRGTCMACAAC-3′), according to the method of Li et al. (2014) [13]. The PCR mixtures contained 1× PCR buffer, 1.5 mM MgCl_2_, 0.2 mM dNTPs, each primer at 0.4 μM, 1.25 U of *Taq* polymerase (Comwin Biotech, Beijing, China), and 1 μL of DNA template in a total volume of 25 μL. PCR was performed with the following program: initial denaturation at 94 °C for 4 min, 35 cycles at 94 °C for 30 s, 52 °C for 30 s, and 72 °C for 30 s, and the reaction batches were incubated at 72 °C for 2 min for terminal elongation.

Using the known MSDIN and *POP* genes from *A. bisporigera* and *G. marginata* as reference models [5, 7], specific primers (shown in Table S1) were designed to obtain target products that were close to the full lengths of the genes according to the flanking sequences of the CDS. The genomic DNA and cDNA of the *Amanita*, *Galerina* and *Lepiota* species were used as templates, and PCR was performed as follows: initial denaturation at 94 °C for 4 min, followed by 32 cycles of denaturation at 94 °C for 30 s, 55–60 °C for 30 s (annealing temperature for each target shown in Table S1), and extension at 72 °C (30 s for an MSDIN gene, 2 min for a *POP* gene), and a final extension at 72 °C for 5 min.

All PCR products were detected by agarose gel electrophoresis and purified using an EasyPure Quick Gel Extraction Kit (Transgen Biotech, Beijing, China). The purified products were ligated into the *pEASY*®-Blunt Zero Cloning Vector (Transgen Biotech, Beijing) and transformed into competent cells. Positive clones to be sequenced were selected using Amp-resistant LB medium and were further verified by colony PCR. Finally, all of the obtained genomic and coding sequences of the genes were used to manually predict the corresponding functions and structures by using DNAMAN 7.0 software.

### Phylogenetic tree construction of MSDIN and *POP* genes

Forty-six coding sequences (CDSs) of MSDIN toxin genes and fifty-eight CDSs of *POP* genes were used for phylogenetic analysis, and their source and GenBank accession numbers are presented in Table 4. These sequences were aligned by using MAFFT v7.374 [37] and then manually adjusted by using BioEdit [38]. HKY + I + G and GTR + I + G were inferred as the best-fit models for the CDSs of the MSDIN and *POP* genes selected according to the AIC in MrModeltest v2.3 [39]. Maximum likelihood (ML) trees with 1000 bootstrap replicates and Bayesian inferences were generated with RAxML v7 [40] and MrBayes v3.1.2 [41], respectively.

## Supplementary information

**Additional file 1: Table S1.** Specific PCR Primers designed for peptide toxins and *POP* genes.

## Data Availability

The mushroom species and related transcriptome data, MSDIN and *POP* genes used in this study has been deposited at GenBank and their accession numbers can be found in Tables 1, 3, 4 and 5.

## Abbreviations

POP: Prolyl oligopeptidase
α-AMA: α-amanitin
β-AMA: β-amanitin
PHD: Phallacidin
PHA: Phalloidin
CDS: Coding sequence
HGT: Horizontal gene transfer
ML: Maximum likelihood

## References

[1] Wieland T (1986). Peptides of poisonous *Amanita* mushrooms.

[2] Enjalbert F, Cassanas G, Rapior S, Chaumont JP (2004). Amatoxins in wood-rotting *Galerina marginata*. Mycologia..

[3] Tang SS, Zhou Q, He ZM, Luo T, Zhang P, Cai Q (2016). Cyclopeptide toxins of lethal amanitas: compositions, distribution and phylogenetic implication. Toxicon..

[4] Walton JD (2018). The cyclic peptide toxins of *Amanita* and other poisonous mushrooms.

[5] Hallen HE, Luo H, Scott-Craiq JS, Walton JD (2007). Gene family encoding the major toxins of lethal *Amanita* mushrooms. Proc Nati Acad Sci USA..

[6] Pulman JA, Childs KL, Sgambelluri RM, Walton JD (2016). Expansion and diversification of the MSDIN family of cyclic peptide genes in the poisonous agarics *Amanita phalloides* and *A bisporigera*. BMC Genomics.

[7] Luo H, Hallen HE, Scott-Craig JS, Walton JD (2012). Ribosomal biosynthesis of α-amanitin in *Galerina marginata*. Fungal Genet Biol.

[8] Luo H, Cai Q, Lüli YJ, Li X, Sinha R, Hallen HE (2018). The MSDIN family in amanitin-produing mushrooms and evolution of the prolyl oligopeptidase genes. IMA Fungus.

[9] Lüli YJ, Cai Q, Chen ZH, Sun H, Zhu XT, Li X (2019). Genome of lethal *Lepiota venenata* and insights into the evolution of toxin-biosynthetic genes. BMC Genomics.

[10] Walton JD, Hallen HE, Luo H (2010). Ribosomal biosynthesis of the cyclic peptide toxins of *Amanita* mushrooms. Biopolymers..

[11] Luo H, Hallen HE, Scott-Craig JS, Walton JD (2010). Colocalization of amanitin and a candidate toxin-processing prolyl oligopeptidase in *Amanita* basidiocarps. Eukaryot Cell.

[12] Luo H, Hong SY, Sgambelluri RM, Angelos E, Li X, Walton JD (2014). Peptide macrocyclization catalyzed by a prolyl oligopeptidase involved in α-amanitin biosynthesis. Chem Biol.

[13] Li P, Deng WQ, Li TH (2014). The molecular diversity of toxin gene families in lethal *Amanita* mushrooms. Toxicon..

[14] Yang ZL (2015). Atlas of the Chinese species of Amanitaceae.

[15] Cui YY, Cai Q, Tang LP, Liu JW, Yang ZL (2018). The family Amanitaceae: molecular phylogeny, higher-rank taxonomy and the species in China. Fungal Divers.

[16] Cai Q, Tulloss RE, Tang LP, Tolgor B, Zhang P, Chen ZH (2014). Multi-locus phylogeny of lethal amanitas: implications for species diversity and historical biogeography. BMC Evol Biol.

[17] Zhang P, Chen ZH, Xiao B, Tolgor B, Bao HY, Yang ZL (2010). Lethal amanitas of East Asia characterized by morphological and molecular data. Fungal Divers.

[18] Li HJ, Xie JW, Zhang S, Zhou YJ, Ma PB, Zhou J (2015). Amanita subpallidorosea, a new lethal fungus from China. Mycol Prog.

[19] Cai Q, Cui YY, Yang ZL (2016). Lethal *Amanita* species in China. Mycologia..

[20] Cai Q, Chen ZH, He ZM, Luo H, Yang ZL (2018). *Lepiota venenata*, a new species related to toxic mushroom in China. J Fungal Res.

[21] Clarke DB, Lloyd AS, Robb P (2012). Application of liquid chromatography coupled to time-of-flight mass spectrometry separation for rapid assessment of toxins in *Amanita* mushrooms. Anal Methods.

[22] Xue JH, Wu P, Chi YL, Xu LX, Wei XY (2011). Cyclopeptides from *Amanita exitialis*. Nat Prod Bioprospect.

[23] Li P, Deng WQ, Li TH (2014). Molecular cloning of *α-amanitin* and characterization of its expression pattern in different parts and development stages of *Amanita exitialis* fruitbody. Mycol Prog.

[24] Zhang CH, Zou JP, Deng WQ, Li TH, Jiang ZD (2018). Molecular cloning and expression pattern of *AePOPB* involved in the α-amanitin biosynthesis in *Amanita exitialis* fruiting body. Gene..

[25] Li P, Deng WQ, Li TH, Song B, Shen YH (2013). Illumina-based de novo transcriptome sequencing and analysis of *Amanita exitialis* basidiocarps. Gene..

[26] He Y, Deng WQ, Zhang CH, Li TH (2019). Diveristy of the MSDIN family in *Amannita rimosa*. Toxicon..

[27] Crooks GE, Hon G, Chandonia JM, Brenner SE (2004). WebLogo: a sequence logo generator. Genome Res.

[28] Sgambelluri RM, Smith MO, Walton JD (2018). Versatility of prolyl oligopeptidase B in peptide macrocyclization. ACS Synth Biol.

[29] Yilmaz I, Bakirci S, Akata I, Bayram R, Kaya E (2015). Toxin content and toxicological significance in different tissues and development stages of *Lepiota brunneoincarnata* mushroom. Toxin Rev.

[30] Wei JH, Wu JF, Chen J, Wu BD, He ZM, Zhang P (2017). Determination of cyclopeptide toxins in *Amanita subpallidorosea* and *Amanita virosa* by high-performance liquid chromatography coupled with high-resolution mass spectrometry. Toxicon..

[31] Keeling PJ, Palmer JD (2008). Horizontal gene transfer in eukaryotic evolution. Nat Rev Genet.

[32] Andersson JO (2009). Gene transfer and diversification of microbial eukaryotes. Annu Rev Microbiol.

[33] Richards TA, Leonard G, Soanes DM, Talbot NJ (2011). Gene transfer into the fungi. Fungal Biol Rev.

[34] Richards TA, Soanes DM, Jones MDM, Vasieva O, Leonard G, Paszkiewica K (2011). Horizontal gene transfer facilitated the evolution of plant parasitic mechanisms in the oomycetes. Proc Nati Acad Sci USA.

[35] Savory F, Leonard G, Richards TA (2015). The role of horizontal gene transfer in the evolution of the oomycetes. PLoS Pathog.

[36] Dhillon B, Feau N, Aerts AL, Beauseigle S, Bernier L, Foster A (2015). Horizontal gene transfer and gene dosage drives adaptation to wood colonization in a tree pathogen. Proc Nati Acad Sci USA..

[37] Katoh K, Standley DM (2016). A simple method to control over-alignment in the MAFFT multiple sequence alignment program. Bioinformatics..

[38] Hall TA (1999). BioEdit: a user-friendly biological sequence alignment editor and analysis program for windows 95/98/NT. Nucleic Acids Sym Ser.

[39] Nylander JAA (2004). MrModeltest v2.2 Uppsala: evolutionary biology Centre, Uppsala University.

[40] Stamatakis A (2006). RAxML-VI-HPC: maximum likelihood-based phylogenetic analyses with thousands of taxa and mixed models. Bioinformatics..

[41] Ronquist F, Huelsenbeck JP (2003). MrBayes 3: Bayesian phylogenetic inference under mixed models. Bioinformatics..

