# Supplementary material for: Diversity of MSDIN family members in amanitin-producing mushrooms and the phylogeny of the MSDIN and prolyl oligopeptidase genes
Source: BMC Genomics. 2020 Jun 26;21:440. doi: 10.1186/s12864-020-06857-8 (PMC7318481; doi:10.1186/s12864-020-06857-8)
Supplement: Supplementary file 1 — Additional file 1: Table S1. Specific PCR Primers designed for peptide toxins and POP genes. [file 12864_2020_6857_MOESM1_ESM.docx]

**Table S1** Specific PCR Primers designed for peptide toxins and *POP* genes.

| **Primer name** | **Sequence (5’→3’)** | **Annealing temp (°C)** | **Target gene** |
| --- | --- | --- | --- |
| UαAMA-F | GAACCCAACTCCAATACCTCTA | 55 | *Ae*, *Asubp*, *Av*_*α-AMA* |
| UαAMA-R | TTCACTACAACACGGATCGTCG |  |  |
| Apa-αAMA-F | ACCCAACTCCAATACCTCTCTC | 55 | *Apa*_*α-AMA* |
| Apa-αAMA-R | GCATATTGACTACAACGCAGAT |  |  |
| Ar-αAMA-F | ACCCAACTCCAATACTTCTC | 55 | *Ar*_*α-AMA* |
| Ar-αAMA-R | CACATATCGTCTACGTGGGT |  |  |
| Gm-αAMA1-F | CAACCGCAAACTCACTTAA | 53 | *Gm*_*α-AMA1* |
| Gm-αAMA1-R | ACACTGAGGATCATCAACGT |  |  |
| Gm-αAMA2-F | CTATCTCATCGGCTCTGTTC | 53 | *Gm*_*α-AMA2* |
| Gm-αAMA2-R | TCAAACAGTAACGATTGTCG |  |  |
| Gs-αAMA1-F | GCTACTCGTCTCCCAATCT | 53 | *Gs*_*α-AMA1* |
| Gs –αAMA1-R | TTACAACACTGGAATCATCG |  |  |
| Gs –αAMA2-F | ATGCAGTTCATGCCAGAGGA | 53 | *Gs*_*α-AMA2* |
| Gs –αAMA2-R | TGGCGTCGCAACTTGATAAT |  |  |
| Lb-αAMA1-F | TAAATCGACTGCTGTTCAGG | 52 | *Lb*_*α-AMA1* |
| Lb-αAMA1-R | CATCGCAGATAAACAACATG |  |  |
| Lb-αAMA2-F | ATCACAATGGACGCAAACTC | 54 | *Lb*_*α-AMA2* |
| Lb-αAMA2-R | CGAGTCCAACAAGGGAACAT |  |  |
| Lv-αAMA1-F | CGAACCCCATCACTCTTACT | 54 | *Lv*_*α-AMA1* |
| Lv-αAMA1-R | CATTCAACATAACATCGCAG |  |  |
| Lv-αAMA2-F | CGCACTCAGACTCATTACTC | 54 | *Lv*_*α-AMA2* |
| Lv-αAMA2-R | AGTAAACGAGTCCAACAAGG |  |  |
| Ae-βAMA-F | CAAGACCTCTAAACCTCCTCC | 54 | *Ae*_*β-AMA* |
| Ae-βAMA-R | ATCGACGTGGGTTTTGTATT |  |  |
| Af-βAMA-F | AAACCTCCAATACCTCTAAACC | 54 | *Af*_*β-AMA* |
| Af-βAMA-R | CAGATCATCGACGTGGATT |  |  |
| Am-βAMA-F | TCAGCACCCAACTCCCATTC | 56 | *Am*_*β-AMA* |
| Am-βAMA-R | TAAATGAGACAACGGGTCCG |  |  |
| Apa-βAMA-F | CTTTCAAACCTCCAAGAACC | 54 | *Apa*_*β-AMA* |
| Apa-βAMA-R | CTCCCGCTAACAAGAAACAC |  |  |
| Ar-βAMA-F | TCTTTCGAACATCTAACACCTC | 54 | *Ar*_*β-AMA* |
| Ar-βAMA-R | CACTTTGACTACAACGTGGGT |  |  |
| Asubp-βAMA-F | TCCAAAGACCTCTAAACCTC | 54 | *Asubp*_*β-AMA* |
| Asubp-βAMA-R | ATAACACTCCCGCTAACAAG |  |  |
| UPHA-F | GACCTCTGCTCTAAATCACA | 55 | *Ae*_*PHA*, *Ae*_“*AWLTDCP*”  and *Asubj*_*PHA* |
| UPHA-R | TGCATTCCATAGTACAACGC |  |  |
| UPHD-F | AATTCTACCTGTCAGAAACCTC | 54 | *Asubj*_*PHD*  *Asubp*_“*AWLVTCP*” |
| UPHD-R | AGCACAAATCATTCATGTGG |  |  |
| Af-PHA-F | CAGCTCTAAATCACAATGTCTG | 54 | *Af*_*PHA* |
| Af-PHA-R | AAGGACAGATCATTTACGTGG |  |  |
| Af-PHD-F | TCAACCAACCGATATGTCTG | 54 | *Af*_*PHD* |
| Af-PHD-R | CACAGTACAACAAACATCATCG |  |  |
| Apa-PHA-F | GAACTTGAACCCTCTAAATCAC | 52 | *Apa*_*PHA* |
| Apa-PHA-R | AAGTTTATTGCGGTCCATG |  |  |
| Apa-UPT-F | CTCAGAATCCTATCAACCGC | 53 | *Apa*_“*AWLMTCP*” |
| Apa-UPT-R | TCATCGACGTGGGTTTTAT |  |  |
| Ar-UPT-F | TATCGTCTCCGTTGTCAGG | 54 | *Ar*_“*AWLECP*” |
| Ar-UPT-R | GTACAGCGCAGATCATTCA |  |  |
| Asubp-PHD-F | ACCTCTTTCAACCGCTATGT | 53 | *Asubp*_*PHD* |
| Asubp-PHD-R | AACATCATCGACGTGGGTT |  |  |
| Asubp-UPT-F | CACTCAGCTTCACTAAATCACA | 54 | *Asubp*_“*AWITDCP*” |
| Asubp-UPT-R | GGAGATCATTTACGTGGGTT |  |  |
| Ae-POPA-F | GAACGCCTTCGCTCTGCTCT | 58 | *Ae*_*POPA* |
| Ae-POPA-R | TGTCGTAGAATGTAGCCCAAAC |  |  |
| Ae-POPB-F | TCCTCCTACCCGTCGTTCTG | 58 | *Ae*_*POPB* |
| Ae-POPB-R | GATTTGGCAGCCTAGTCAACC |  |  |
| Af-POPA-F | CCGTTCGGACCACATAGACAC | 58 | *Af*_*POPA* |
| Af-POPA-R | TACCTGGAATCGTGAGTGTCGT |  |  |
| Af-POPB-F | CACAGTTATCCTCCTACTCGTCG | 58 | *Af*_*POPB* |
| Af-POPB-R | CGTGTAAACAGTGTTTTGGCCTAG |  |  |
| Am-POPA-F | TACCCTCCTACTCGTCGTTC | 55 | *Am*_*POPA* |
| Am-POPA-R | TGTAGCCCAAACAGTAACAGG |  |  |
| Am-POPB-F | TCCTCACAGTTATCCTCCCA | 50 | *Am*_*POPB* |
| Am-POPB-R | CATTTCATTGACGGCATAGT |  |  |
| Ao-POPA-F | GTCCCAAAGTCCAAGTTCCA | 54 | *Ao*_*POPA* |
| Ao-POPA-R | TCCGTTCACAAATTGGCTTG |  |  |
| Apa-POPA-F | ACACCGTCTCAAATCCAAGC | 55 | *Apa*_*POPA* |
| Apa-POPA-R | GCACAAGTGCAGTTGCGTTA |  |  |
| Apa-POPB-F | CAGTTATCCTCCTACTCGTCG | 55 | *Apa*_*POPB* |
| Apa-POPB-R | GATCTAACAACGTGGCTTCA |  |  |
| Ar-POPA-F | TCGCCGTTCAGACCACATTG | 58 | *Ar*_*POPA* |
| Ar-POPA-R | CCATTTGTCGGCAGCCTCTT |  |  |
| Ar-POPB-F | GCTTCCACCCTTACCACAGA | 58 | *Ar*_*POPB* |
| Ar-POPB-R | CAGCCTAGTCGTCCGTTTGC |  |  |
| Asubp-POPA-F | ACCCAACACGTACCCTCCTA | 60 | *Asubp*_*POPA* |
| Asubp-POPA-R | ACTCATCACAGATTGGCTTGT |  |  |
| Asubp-POPB-F | GGCTCCTCACAGTTATCCTCC | 60 | *Asubp*_*POPB* |
| Asubp-POPB-R | ATTTGACCGCCTAGTCATCC |  |  |
| Gm-POPA-F | AGAGGAATACACCGATGAGAC | 54 | *Gm*_*POPA* |
| Gm-POPA-R | TGAGCTGGATTCTACGATTAG |  |  |
| Gm-POPB-F | CTCCTGGAAATTATCCCTCT | 52 | *Gm*_*POPB* |
| Gm-POPB-R | ATACATTCTATGCCAAGTCCTC |  |  |
| Gs-POPA-F | CCGTGGCTTCCTGATGCTTA | 57 | *Gs*_*POPA* |
| Gs-POPA-R | TCATGCGTCGAGTCCCAGTT |  |  |
| Gs-POPB-F | GACAAATGGACGACTGCTC | 55 | *Gs*_*POPB* |
| Gs-POPB-R | AGCCAGGATCTAGTTCAGGT |  |  |
| Lv-POPA-F | TTCACGCTCATCTACAACGG | 55 | *Lv*_*POPA* |
| Lv-POPA-R | ATCTCCCTGCTTTCACCCAC |  |  |
| Lv-POPB-F | CTCTGCTCGCCGTTCTGAT | 55 | *Lv*_*POPB* |
| Lv-POPB-R | TGAATCGGATGTCTGGTTAGTT |  |  |

*Ae*, *A. exitialis*; *Af*, *A. fuliginea*; *Am*, *A. molliuscula*; *Ao*, *A. oberwinklerana*; *Apa*, *A. pallidorosea*; *Ar*, *A. rimosa*; *Asubf*, *A. subfuliginea*; *Asubj*, *A. subjunquillea*; *Ab*, *A. subpallidorosea*; *Av*, *A. virosa*; *Gm*, *G. marginata*; Gs, *G. sulciceps*; Lv, *L. venenata*, *α-amanitin*, *α-AMA*; *β-amanitin*, *β-AMA*; *PHA*, *phallacidin*; *PHD*, *phalloidin*; POP, prolyl oligopeptidase.
